# The pharmacological mechanism of Chinese herbs effective in treating advanced ovarian cancer: Integrated meta-analysis and network pharmacology analysis

**DOI:** 10.3389/fphar.2022.1040641

**Published:** 2022-11-09

**Authors:** Ze Yang, Xiang Wang, Wei Hong, Shiyi Zhang, Yang Yang, Yongliang Xia, Ruiwen Yang

**Affiliations:** ^1^ The First Clinical Medical College, Zhejiang Chinese Medical University, Hangzhou, China; ^2^ Department of Orthopedics, Tongde Hospital of Zhejiang Province, Hangzhou, China; ^3^ Department of Traditional Chinese Medicine, Neighborhood Good Doctor No. 6 Street Clinic, Hangzhou, China; ^4^ Health Management Center, The First Affiliated Hospital of Zhejiang Chinese Medical University (Zhejiang Provincial Hospital of Chinese Medicine), Hangzhou, China

**Keywords:** advanced ovarian cancer, traditional Chinese medicine, meta-analysis, network pharmacology analysis, review

## Abstract

**Background:** Advanced ovarian cancer (AOC) develops rapidly, adding to difficulties in treatment. Traditional Chinese medicine (TCM) plays a significant role in the treatment of AOC, and so to explore the efficacy and safety of TCM in the treatment of AOC and its effective targets, we performed the following review.

**Methods:** The major databases were searched for randomized controlled trials of TCM for the treatment of AOC. A meta-analysis of the efficacy of Chinese herbs on AOC was conducted using RevMan 5.4 software. Active compounds and target genes were acquired using the TCMSP database. The main targets of AOC were obtained through the GenCards, OMIM, TTD, and DrugBank databases. A protein–protein interaction network carried out on the STRING platform was used to select core genes. The Metascape platform was applied to achieve GO and KEGG enrichment analysis.

**Results:** A total of 24 studies were included. Meta-analysis shows the TCM group improved the overall response rate (OR = 2.71; 95% CI = [2.14, 3.44], Z = 8.25, *p* < 0.00001), overall survival (OR = 2.93, 95% CI = [2.03, 4.24], Z = 5.72, *p* < 0.00001), and progression-free survival (OR = 5.36, 95% CI = [5.03, 5.69], Z = 31.88, *p* < 0.00001) of AOC patients, as well as reducing many adverse events. There were 120 compounds, 246 herb target genes, and 1503 disease targets extracted. The 10 most important components were quercetin, kaempferol, 7-methoxy-2-methyl isoflavone, formononetin, isorhamnetin, hederagenin, stigmasterol, luteolin, 7-O-methylisomucronulatol, and calycosin. The 20 core targets were *TP53*, *STAT3*, *JUN*, *AKT1*, *MAPK3*, *RELA*, *MAPK1*, *ESR1*, *IL6*, *FOS*, *MAPK14*, *TNF*, *CDKN1A*, *RB1*, *CCND1*, *EGFR*, *STAT1*, *MDM2*, *MAPK8*, and *CAV1*. KEGG enrichment analysis showed that there are many pathways directly related to different types of tumors, such as in pathway cancer and prostate cancer.

**Conclusion:** Our article reveals TCM is effective and safe against AOC and that Chinese herbs exert effects on the disease through multi-target, multi-component, and multi-pathway mechanisms.

**Systematic Review Registration:** (www.crd.york.ac.uk/PROSPERO/), identifier (CRD42022369731).

## Introduction

Ovarian cancer (OC) ranks fifth among cancer deaths in women, representing a larger number of deaths than any other cancer type of the female reproductive system, and is connected to the highest number of deaths among gynecological cancers in developed countries (Carioli et al., 2021; Craig et al., 2021).

Due to the complex anatomical structure and endocrine function of ovarian tissue, together with the lack of obvious clinical symptoms in the early stages (Han and Shen, 2015), the onset of OC is insidious (Menon et al., 2018). Most ovarian cancer cases are diagnosed at an advanced stage with a 5-year survival of just 15%–25% (Torre et al., 2018; Lheureux et al., 2019a). The main treatment for advanced ovarian cancer (AOC) is primary debulking surgery, combined with carboplatin and paclitaxel chemotherapy (Colombo et al., 2019). However, up to 80% of patients inevitably develop chemo-resistance and experience relapses, with a median progression-free survival of 12–18 months (Lheureux et al., 2019b). Moreover, many patients cannot tolerate the adverse reactions caused by long-term chemotherapy, which has a serious impact on their prognosis.

In recent years, considerable experience has been accumulated in the use of traditional Chinese medicine (TCM) in the treatment of OC, and with the characteristics of overall conditioning and multi-target intervention, TCM has achieved good results in assisting with chemotherapy (Xu et al., 2015; Wang et al., 2016; Yang, 2020). However, the clinical characteristics of effective herbs for AOC and their components and targets have not been explored before. Therefore, our article aims to evaluate effective herbs in the treatment of AOC through meta-analysis. Moreover, the potential pharmacological mechanism of such effective herbs is explored using a network pharmacology approach.

## Materials and methods

### Meta-analysis of the efficacy and safety of TCM for AOC

#### Database and search strategies

We searched PubMed, Embase, the Cochrane Library, the Chinese Biomedical Literature (CBM) database, the China National Knowledge Infrastructure (CNKI) database, and the Wanfang database for updated articles published from the establishment of each database to 1 August 2022. The search keywords we used included: (“randomized controlled trials as topic” OR “controlled clinical trial*” OR “randomized*” OR “placebo” OR “clinical trial*” OR “controlled trial*“) AND (“decoction” OR “formula” OR “Tang” OR “Traditional Chinese medicine”) AND (“neoplasm metastasis” OR “IV stage” OR “advanced ovarian cancer” OR “metastatic ovarian cancer” OR “ovarian cancer” OR “ovarian neoplasms”).

#### Eligibility criteria

Detailed inclusion and exclusion criteria are shown in Table 1.

**TABLE 1:** Inclusion and exclusion criteria.

PICOS	Inclusion criteria	Exclusion criteria
Participants	1 Age 18 years or older	1 Younger than 18 years
	2 Pathologically diagnosed as AOC, according to the FIGO system (Fédération Internationale de Gynécologie et d’Obstétrique), which considers the extent of tissue involvement, lymph node status, and the magnitude of metastasis. The stage III and stage IV cancers that spread beyond the pelvic cavity are called AOC (11)	2 Unclear diagnosis of AOC
	3 Indications of chemotherapy, exclusion of contraindications of chemotherapy, lack of center, liver, kidney, and other major organs and serious systemic diseases	3 Contraindications of chemotherapy, including lack of center, liver, kidney, and other major organs and serious systemic diseases
	4 Estimated survival ≥ 3 months	4 Estimated survival ＜ 3 months
Intervention	The intervention group was treated with TCM therapy including oral TCM decoction and Chinese patent medicine combined with chemotherapy. Chemotherapy regimens were not restricted	The intervention group was treated with acupuncture, tuina, or acupoint application and other external therapies of Chinese medicine
Comparison	The control group was treated with conventional chemotherapy	The control group was treated with TCM treatment
Outcome	Overall response rate (ORR), overall survival (OS), progression-free survival (PFS), and adverse events (AEs)	Incomplete or unidentified data
Study design	Randomized controlled trial (RCT)	Non-RCTs
Others	None	Duplicate publications, abstracts, reviews, case reports, and letters

#### Study selection and data extraction

Two researchers (ZY and XW) independently searched the articles in the databases. The titles and abstracts were screened according to the inclusion and exclusion criteria, and then the full texts of the remaining articles were screened for a final decision. If there was a disagreement, the third researcher (RWY) would be consulted. The extraction information from the included studies entailed: first author, sample size, mean age or age range, clinical status, common treatment (regimen), TCM interventions, control interventions, duration time, and main outcomes.

#### Assessment of the risk of bias

The risk of bias for all included studies was assessed by two researchers (ZY and XW) independently, based on the Cochrane risk bias tool in the *Cochrane Handbook for Systematic Reviews of Interventions* (Cumpston et al., 2019). The risk bias includes random sequence generation, allocation concealment, blinding of participants and implementers, blinding of outcome assessment, incomplete outcome data, selective reporting, and other potential biases.

#### Statistical analysis

RevMan 5.4 software was employed for analyzing the collected data. The odds ratio (OR) was used as the effect size index for the dichotomous variables, and the mean difference (MD) was used as the effect size index for the continuous variables, with 95% confidence intervals (CI) in forest plots. If heterogeneity existed (*p* ＜ 0.1 or I^2^ > 50%) between the two groups, the random-effects model was adopted. Otherwise, the fixed-effects model was used. Subgroup analyses were performed based on the types of OS and AEs. Publication bias was evaluated visually using funnel plots in RevMan 5.4 software.

### Network pharmacology of effective herbs for AOC

Analysis of the frequency of the presence of herbs in prescriptions extracted from articles was included in the above meta-analysis. Herbs with a frequency greater than 50% were considered effective herbs and were used for subsequent network pharmacology analysis.

#### Screening of active compounds and target genes of effective herbs and the herb–component–target network

The chemical compounds of effective herbs were obtained through the TCMSP database (http://tcmspw.com/tcmsp.php). Active compounds of herbs were selected if their drug-likeness (DL) index ≥ 0.18 and oral bioavailability (OB) ≥ 30%. Then, using the UniProt database (https://www.uniprot.org) to annotate the related target genes, the herb–component–target network of herbs effective against AOC was established by Cytoscape3.7.2 software, with the 10 most important components selected according to their degree value in the network.

#### Screening of disease targets

High correlation AOC-related targets were extracted by searching the keyword term “advanced ovarian cancer” from the following public databases: DrugBank (https://www.drugbank.ca/), GeneCards (https://www.genecards.org/), OMIM (http://omim.org/), and TTD (http://db.idrblab.net/ttd/).

#### Acquisition of intersectional genes and construction of protein–protein interaction

Genes intersecting the target genes of effective herbs and the targets of AOC were extracted using a Venn diagram made on a website (https://bioinfogp.cnb.csic.es/tools/venny/index.html). The intersectional genes were then imported into the STRING platform (https://stringdb.org/). Homo was the species, a score > 0.9 was set, and the independent target protein nodes were hidden. The results were exported in TSV format and then imported into Cytoscape3.7.2. The CytoNCA plugin calculated the following four parameters: betweenness centrality (BC), closeness centrality (CC), degree centrality (DC), and eigenvector centrality (EC), and the first 20 core genes with higher than average values were extracted.

#### GO and KEGG enrichment analysis

The KEGG pathway enrichment analysis and GO enrichment analysis were obtained based on the Metascape database (https://metascape.org/gp/index.html). The GO enrichment analysis included the biological process (BP), molecular function (MF), and cellular component (CC) analysis. The top 10 records with q value < 0.05 in terms of GO enrichment analysis and the top 20 KEGG pathways were extracted, with the latter imported into Cytoscape3.7.2 for visualization of the target–pathway network of effective prescription herbs against AOC.

## Results

### Results of meta-analysis

#### Search results

**FIGURE 1:**
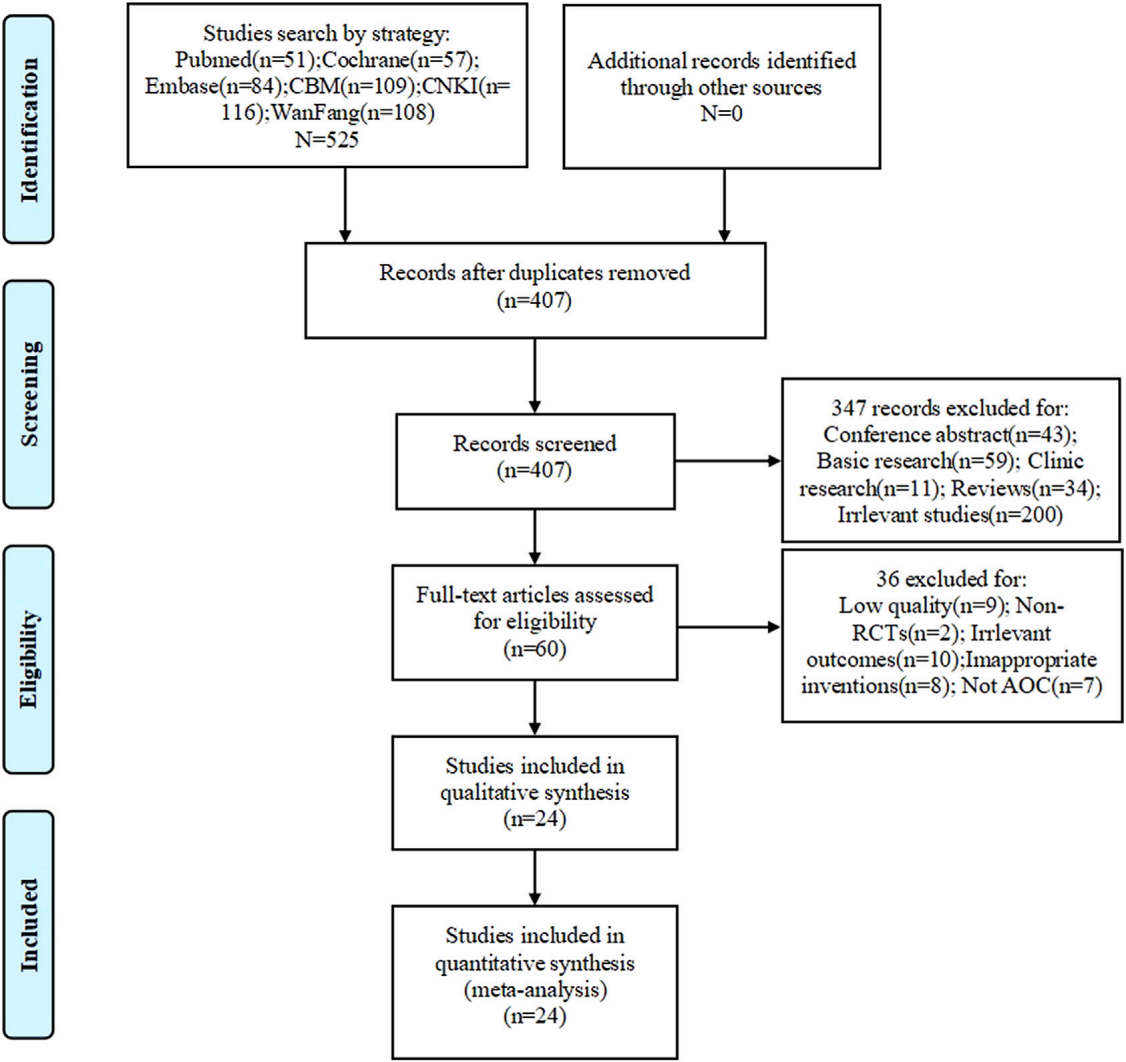
PRISMA flow diagram of the study selection process.

All 525 records were obtained by searching the databases, of which 118 records were excluded for duplication. After screening titles and abstracts, a further 347 items were removed because they were conference abstracts, basic research, clinical research, reviews, or irrelevant. Sixty articles were reviewed for full-text evaluation, among which 36 were excluded for having low quality, irrelevant outcomes, inappropriate inventions, non-RCTs, or not being related to AOC. Finally, 24 were included in this meta-analysis (Pei, 2010; Chen, 2012; Liang, 2013; Chen et al., 2014; Jin and Kong, 2015; Zhang, 2016; Zhao et al., 2016; Chen and Hua, 2017; Jia, 2017; Yang, 2017; Zhou, 2017; Hou and Wu, 2018; Pan, 2018; Zhang et al., 2018; Fang et al., 2019; Hu, 2019; Ren and Feng, 2019; Li et al., 2020; Zhou et al., 2020; Dai and Liu, 2021; Li et al., 2021; Yang and Mi, 2021; Huang et al., 2022; Wang et al., 2022). No further study was identified by manual search. The flow diagram of studies selection is shown in Figure 1, while the main characteristics of the 24 included articles are provided in Table 2.

**TABLE 2:** Characteristics of the 24 studies included in the meta-analysis.

Study	Sample size (T/C)	Mean age or age range		Clinical status	Common treatment (regimen)	TCM intervention	Control intervention	Main outcome
		T	C					
Chen2012	27/25	52.36 ± 6.48	51.12 ± 6.20	KPS＞60	TP	TCM experience formula	No additional Tx	②
Chen2017	50/50	66.52 ± 6.01	66.35 ± 5.96	KPS ≥ 60	Cisplatin	TCM	No additional Tx	①②③
Chen2014	43/34	48.8 ± 4.1	52.6 ± 3.1	Not mentioned	Taxol	Fuzheng Xiaoliu decoction	No additional Tx	①③
Dai2021	35/35	52.15 ± 6.83	52.07 ± 6.79	KPS＞60	TC	Guizhi Fuling Wan	No additional Tx	①③
Fang2019	56/56	51.52 ± 7.38	52.03 ± 7.61)	KPS ≥ 60	TC	Wenyang Yiqi Jianpi decoction	No additional Tx	①②③
Hou2018	38/32	52.27 ± 7.50	53.69 ± 7.67	Not mentioned	TP	TCM	No additional Tx	②
Hu2019	40/40	58.41 ± 3.78	57.78 ± 3.81	KPS ＞ 60	TC	Jiandu Yiai decoction	No additional Tx	①③
Huang2022	40/40	52.12 ± 7.65	51.53 ± 7.40	KPS ≥ 60	TC	Wenyang Yiqi Jianpi decoction	No additional Tx	①③
Jia2017	42/42	53.55 ± 4.51	39.7 ± 15.4	Not mentioned	TP	TCM	No additional Tx	②
Jin2015	43/34	51.5 ± 4.3	50.7 ± 3.6	Not mentioned	Taxol	Fuzheng Xiaoliu decoction	No additional Tx	①③
Li2020	153/146	54 ± 9	56 ± 9	KPS ≥ 70	Paclitaxel and platinum-based chemotherapy	Yiqi Huoxue Jiedu decoction	Placebo	④
Li2021	54/54	60.12 ± 6.78	60.24 ± 6.82	KPS ≥ 60	TC	Yiqi Huoxue Jiedu decoction	No additional Tx	①③
Liang2013	60/60	24–72	22–73	KPS ≥ 60	TP	Zengmian Yiliu decoction	No additional Tx	③
*Pan*2018	28/28	62.13 ± 3.67	61.79 ± 3.50	Not mentioned	Docetaxel + cisplatin	Yiqi Jianpi Yangxue decoction	No additional Tx	③
Pei2011	35/35	52.23 ± 3.46	51.77 ± 2.81	KPS ≥ 70	Basic chemotherapy	Lichong decoction	No additional Tx	③
Ren2019	44/44	51.09 ± 8.32	50.19 ± 6.77	Not mentioned	TP	Fuzheng Quji decoction	No additional Tx	①③
Wang2022	46/46	49	52	KPS ＞ 60	Basic chemotherapy + docetaxel	Yiqi Yangyin decoction	No additional Tx	①②③
Yang2017	23/23	50.31 ± 10.36	51.31 ± 9.74	Not mentioned	Basic chemotherapy	Huoxue Jiedu decoction	No additional Tx	①③
Yang2021	56/42	52.23 ± 8.97	51.64 ± 8.56	Not mentioned	TC	Compound Daqiqi Decoction	No additional Tx	①
Zhang2018	46/46	52.03 ± 9.12	51.80 ± 9.24	Not mentioned	TP	TCM	No additional Tx	①②
Zhang2016	36/36	59.47 ± 9.03	58.94 ± 8.63	KPS ＞ 30	TP	TCM	No additional Tx	①③
Zhao2016	24/24	55	Not Mentioned	TC	Taohong Siwu decoction	No additional Tx	①	
Zhou2017	30/30	55.13 ± 2.53	54.68 ± 2.58	Not mentioned	TP	Jianpi Jiedu Sanjie decoction	No additional Tx	①③
Zhou2020	48/48	54.78 ± 5.48	55.13 ± 5.51	KPS ＞ 60	TC	Lichong decoction	No additional Tx	① ③

#### Assessment of risk of bias

**FIGURE 2:**
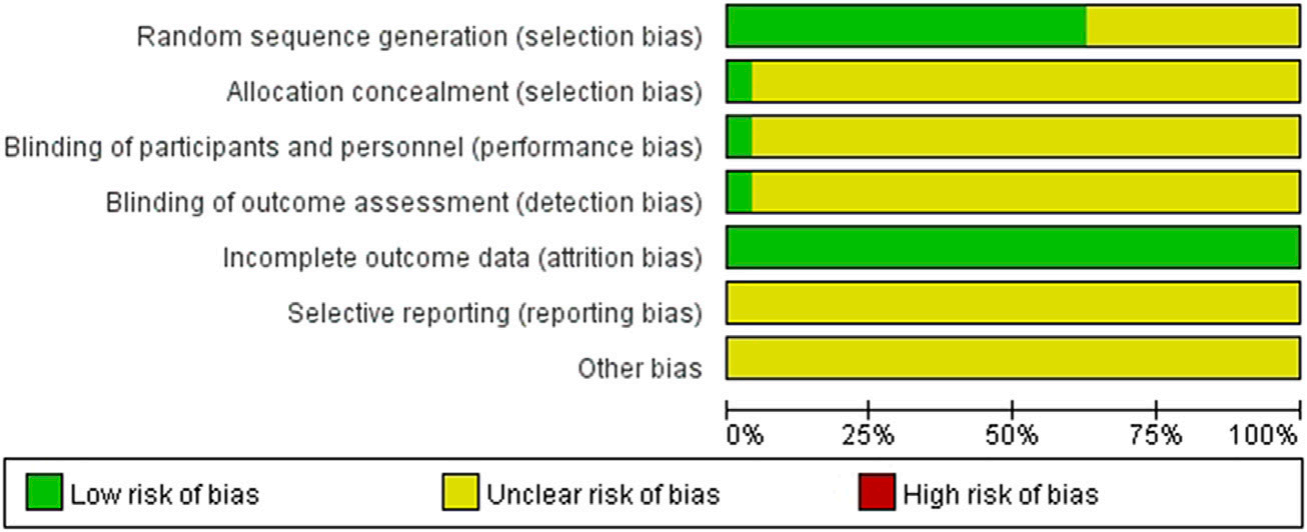
Risk of bias graph.

**FIGURE 3:**
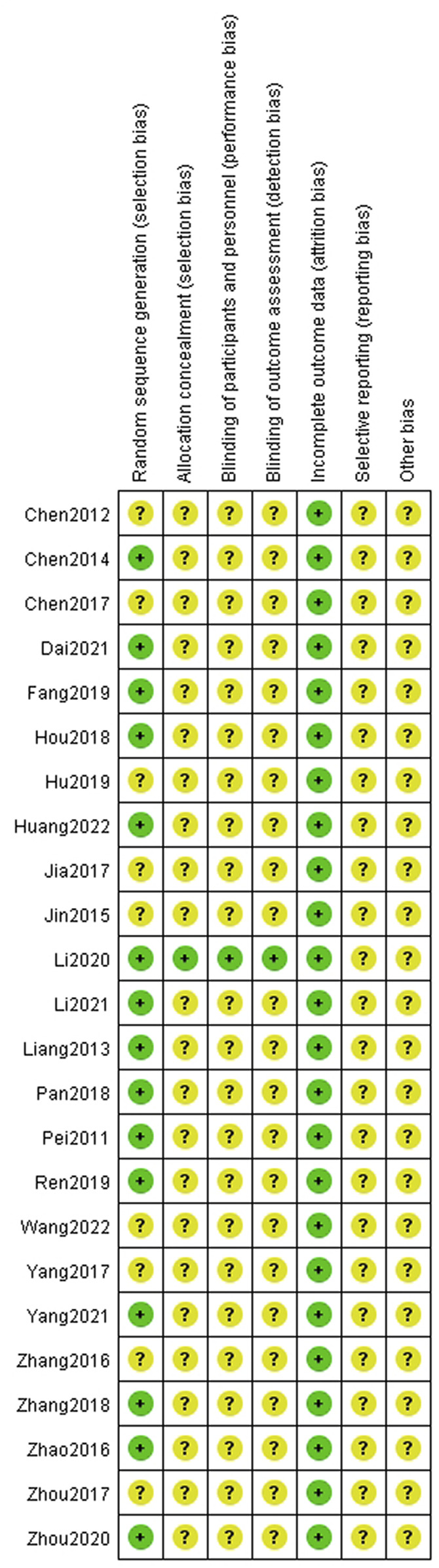
Risk of bias summary.

The results of the assessment of risk of bias are shown in Figures 2, 3. All articles employed randomization, and 15 studies using the means of random number table were considered to have a low risk of bias. Those studies that did not mention specific random methods were considered to possess an unclear risk of bias. One study (Li et al., 2020) which mentioned allocation concealment, blinding of participation, and outcome assessment, was considered low risk; others did not mention whether allocation concealment and blinding of participation was adopted or not, and were thus considered to have an unclear risk of bias. All studies were completed with data and considered low risk. Selective reporting and other biases in the included articles resulted in an unclear risk of bias, since these potential biases were not acknowledged in the articles.

#### Overall response rate

**FIGURE 4:**
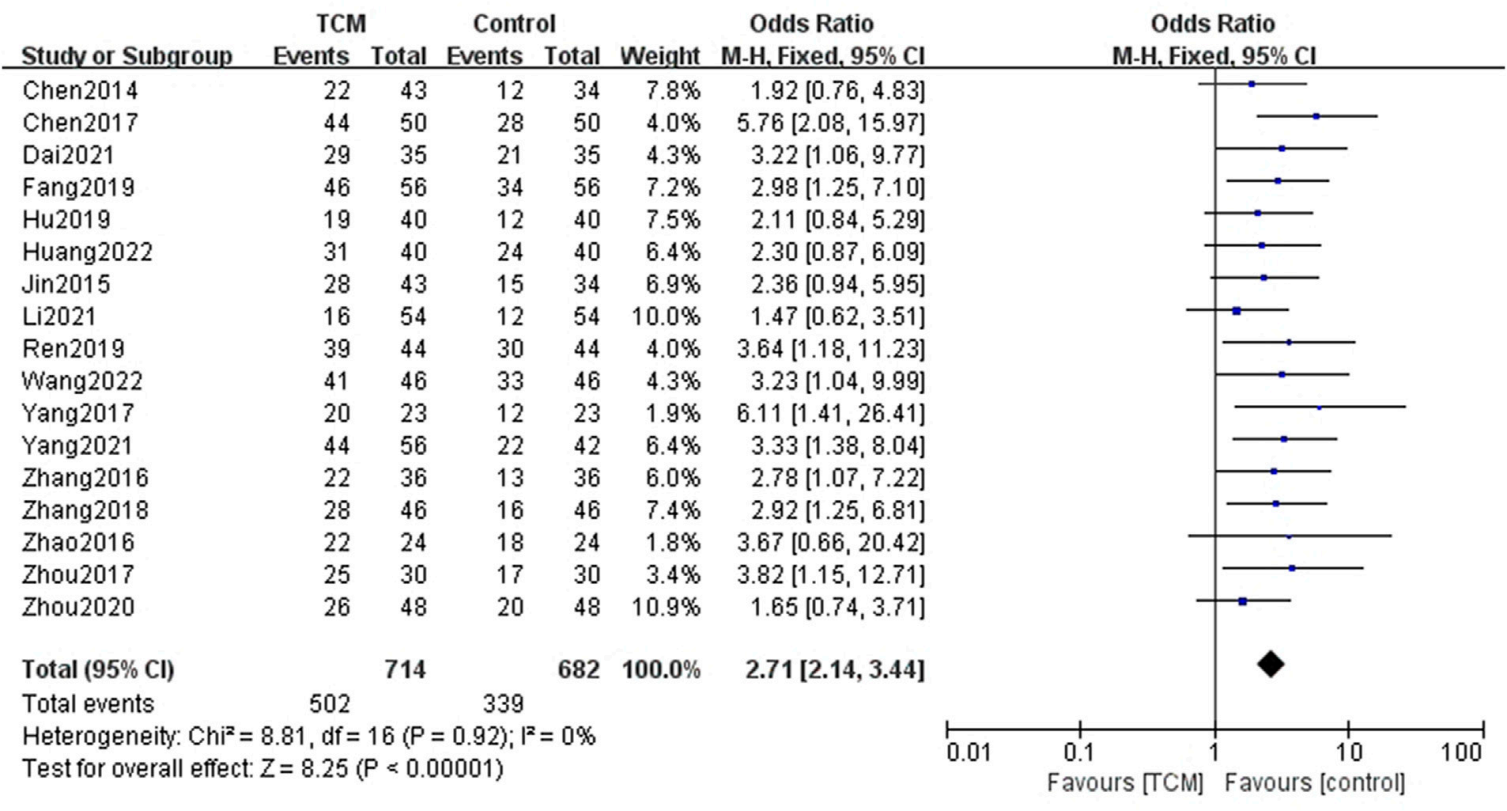
Forest plots for comparison of ORR between TCM group and control group.

Data from 17 related studies indicating overall response rate were synthesized. In AOC patients, the ORR in the TCM combined with the chemotherapy group was significantly better than in the other group (OR = 2.71; 95% CI = [2.14,3.44], Z = 8.25, *p* < 0.00001) (Figure 4).

#### Overall survival

**FIGURE 5:**
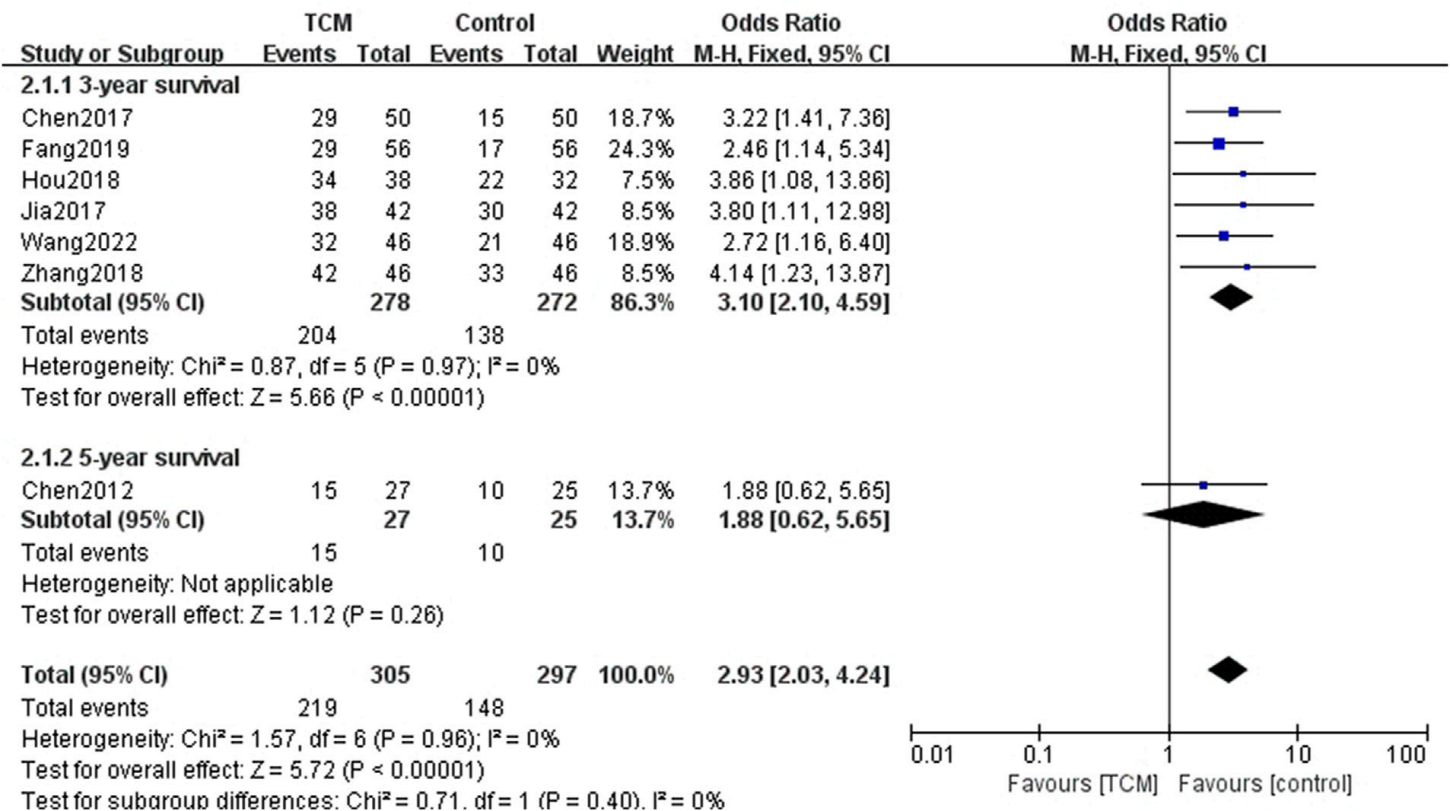
Forest plots and subgroup analysis for comparison of OS between TCM group and control group.

As shown in Figure 5, seven related studies employed a fixed-effects model for the pool of data reflecting overall survival (OS). The pooled results show that TCM combined with chemotherapy is beneficial in improving OS (OR = 2.93, 95% CI = [2.03, 4.24], Z = 5.72, *p* < 0.00001).

There were significant differences in 3-year survival between the TCM group and the control group in the subgroup analysis (OR = 3.10, 95% CI = [2.10, 4.59], Z = 5.66, *p* < 0.00001). However, there were no significant differences in 5-year survival (OR = 1.88, 95% CI = [0.62, 5.65], Z = 1.12, *p* < 0.00001).

#### Progression-free survival

**FIGURE 6:**
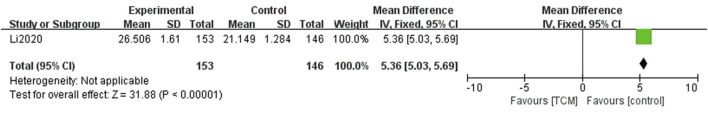
Forest plots and subgroup analysis for comparison of PFS between TCM group and control group.

Only one study reported the outcome of progression-free survival (PFS). This result indicated that the PFS in the TCM group was significantly higher than in the control group (OR = 5.36, 95% CI = [5.03, 5.69], Z = 31.88, *p* < 0.00001) (Figure 6).

#### Adverse events

**FIGURE 7:**
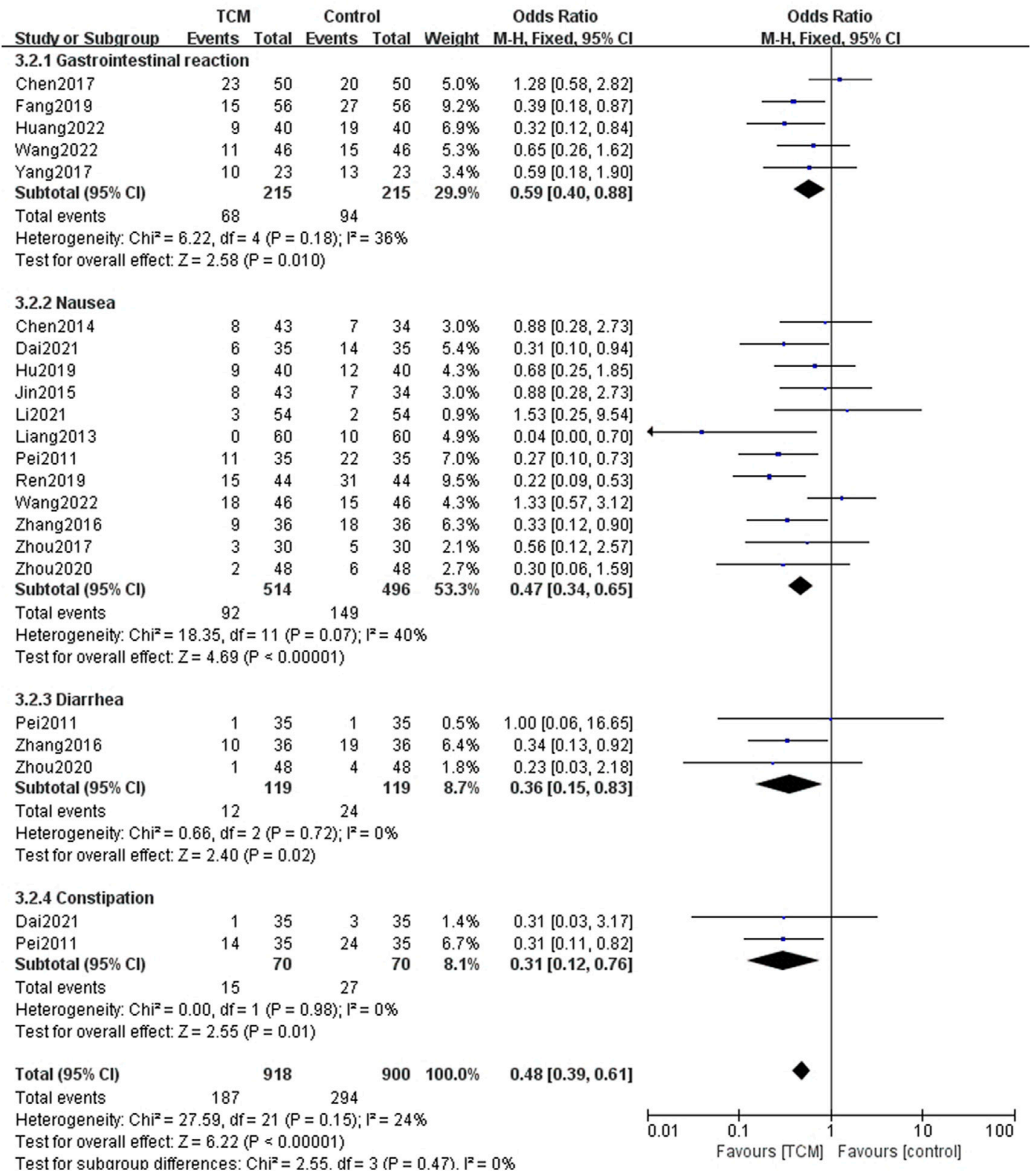
Forest plots and subgroup analysis for gastrointestinal adverse event.

Concerning adverse events, 17 of the included articles reported these. The most common type of adverse event reported was a gastrointestinal reaction. There were 16 studies reporting this, in which 5 reported gastrointestinal reactions generally, while others reported specific reactions, such as nausea, diarrhea, and constipation. The pooled results of 12 studies showed that the incidence of nausea in the TCM group was lower than that in WM group (OR = 0.47, 95% CI = [0.34, 0.65], Z = 4.69, *p* < 0.00001). Three studies reported the occurrence of diarrhea in the two groups, while the TCM group also did better in reducing the incidence of nausea (OR = 0.36, 95% CI = [0.15, 0.83], Z = 2.40, *p* = 0.02). Only two studies mentioned constipation, and in the TCM group, the incidence of constipation was significantly lower (OR = 0.31, 95% CI = [0.12, 0.76], Z = 2.55, *p* = 0.01). The results showing gastrointestinal reaction are in Figure 7.

**FIGURE 8:**
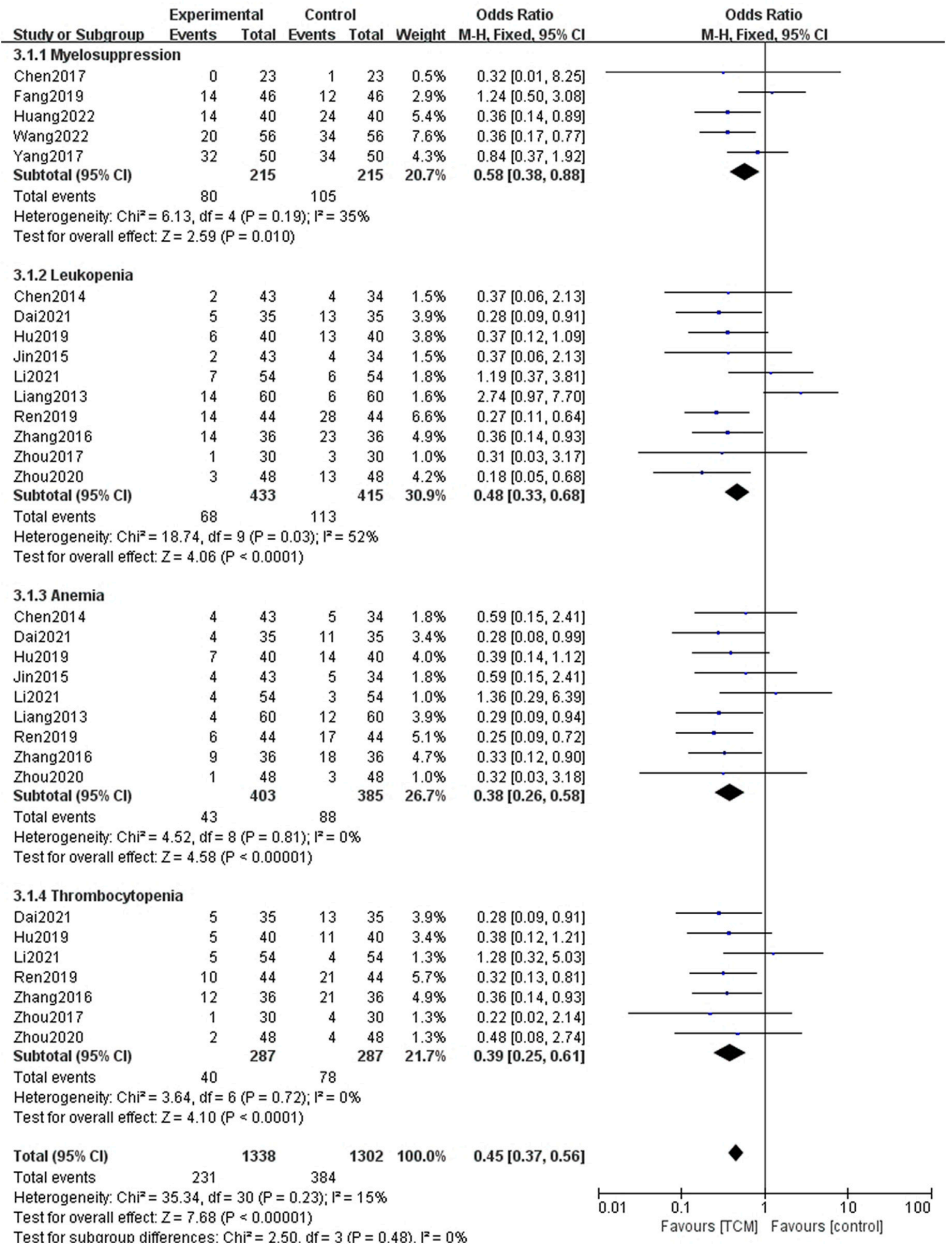
Forest plots and subgroup analysis for myelosuppression event.

The adverse event of myelosuppression was mentioned in 15 studies, 5 of them reporting myelosuppression generally, with the remainder reporting an instance of specific myelosuppression, such as leukopenia, anemia, or thrombocytopenia, as shown in Figure 8. TCM intervention can reduce the occurrence of myelosuppression (OR = 0.45, 95% CI = [0.37,0.56], Z = 7.68, *p* ＜0.0001). Ten trials reported the adverse event of leukopenia between the two groups, and the results showed that the TCM group did better in decreasing the incidence of leukopenia (OR = 0.48, 95% CI = [0.33,0.68], Z = 4.06, *p* ＜ 0.0001). In addition, 9 studies reported the occurrence of anemia. The pooled results showed that there were significant differences in the occurrence of anemia between the two groups (OR = 0.38, 95% CI = [0.26,0.58], Z = 4.58, *p* ＜ 0.00001). Subgroup analysis of seven studies showed that TCM intervention can reduce the incidence of thrombocytopenia (OR = 0.39, 95% CI = [0.25,0.61], Z = 4.10, *p* ＜ 0.0001).

**FIGURE 9:**
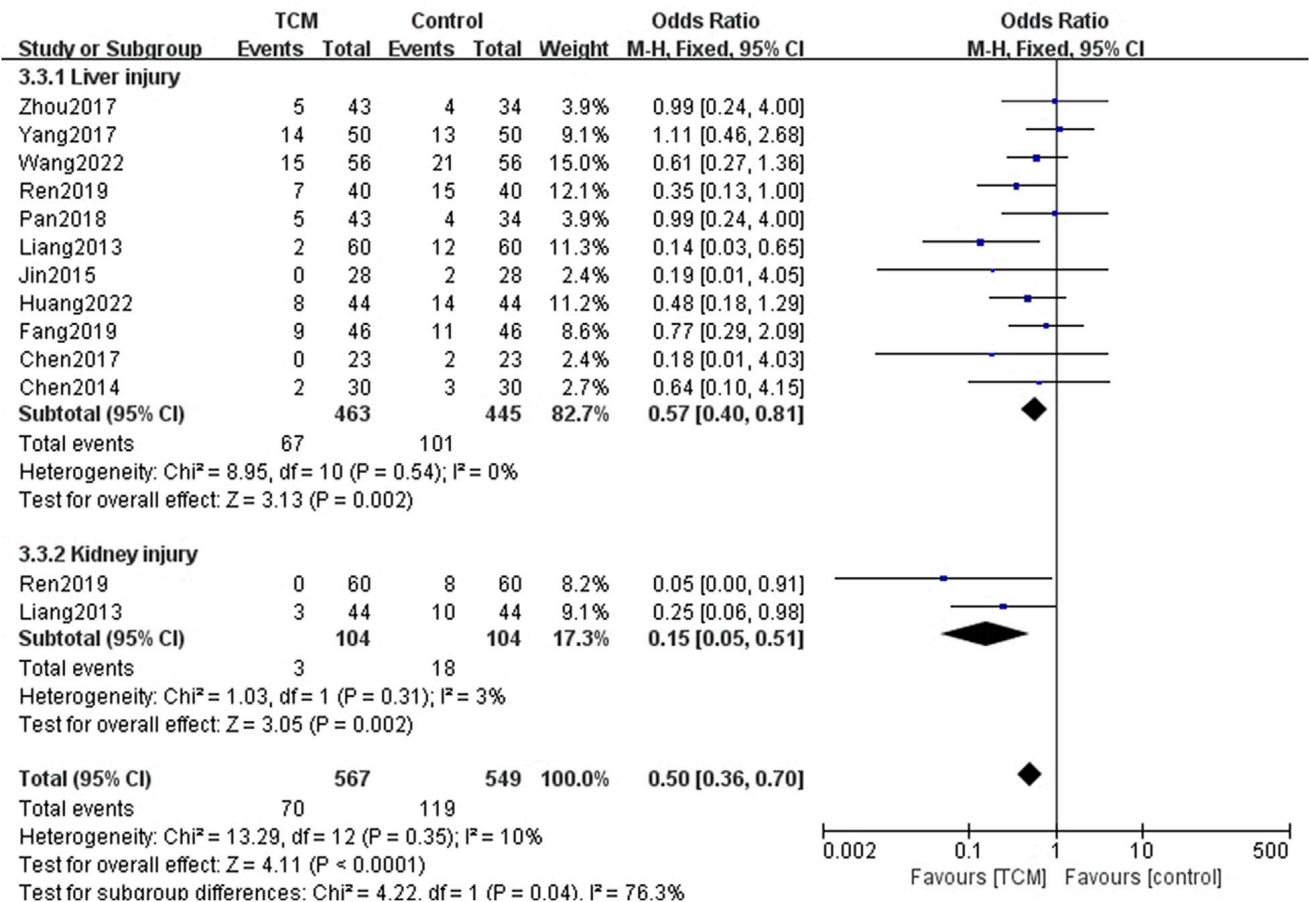
Forest plots and subgroup analysis for liver and kidney injury event.

Liver and kidney injury were also common when treating AOC. Liver injury in 11 trials and kidney injury in 2 studies were reported. Subgroup analysis showed both liver injury (OR = 0.57, 95% CI = [0.40,0.81], Z = 3.13, *p* = 0.002) and kidney injury (OR = 0.50, 95% CI = [0.36,0.70], Z = 3.05, *p* = 0.002) were reduced in the TCM group (Figure 9).

**FIGURE 10:**
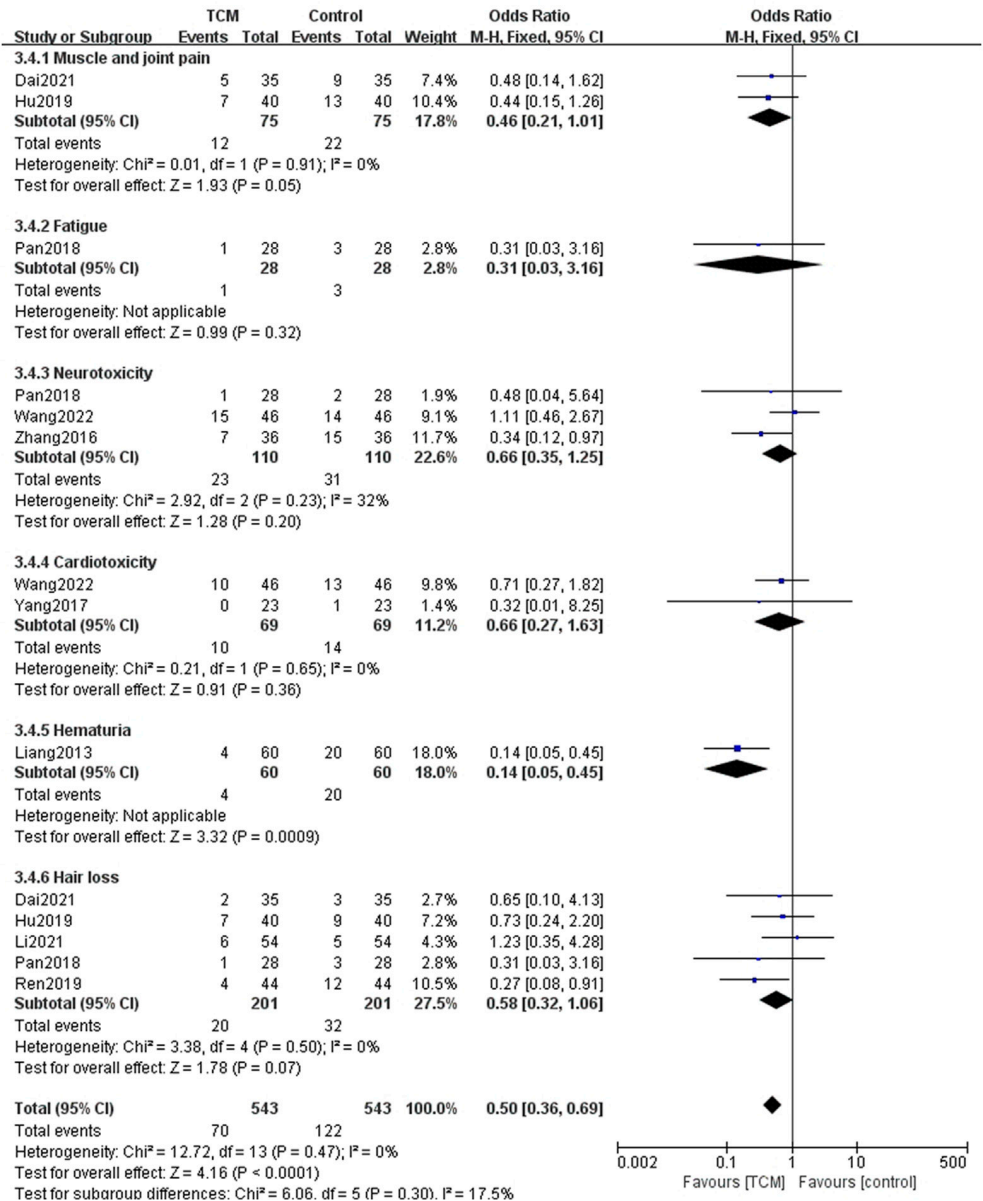
Forest plots and subgroup analysis for other adverse events.

Some other adverse events were also mentioned. Among them, compared with the control group, the TCM group had benefit in reducing hematuria (OR = 0.14, 95% CI = [0.05,0.45], Z = 3.32, *p* = 0.0009), but no obvious effect on decreasing the incidence of muscle and joint pain (OR = 0.46, 95% CI = [0.21,1.01], Z = 1.93, *p* = 0.05), fatigue (OR = 0.31, 95% CI = [0.03,3.16], Z = 0.99, *p* = 0.32), neurotoxicity (OR = 0.66, 95% CI = [0.35,1.25], Z = 1.28, *p* = 0.20), cardiotoxicity (OR = 0.66, 95% CI = [0.27,1.63], Z = 0.91, *p* = 0.36), and hair loss (OR = 0.58, 95% CI = [0.32,1.06], Z = 1.78, *p* = 0.07) (Figure 10).

#### Publication bias

**FIGURE 11:**
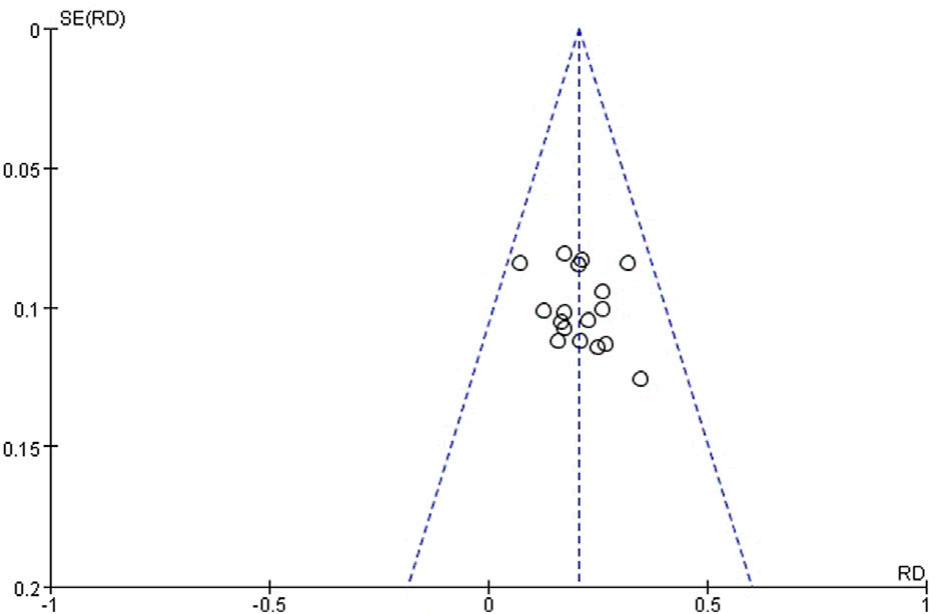
Funnel plot about ORR.

Funnel plots of ORR were adopted to assess publication bias. It was apparent from the funnel plot (Figure 11), that the result was nearly symmetrical, which indicates no significant publication bias existed.

### Results of network pharmacology analysis

#### Effective herbs extraction

We analyzed the frequency of herbs appearing in the twenty different TCM formulas mentioned in the included articles. Sorted according to their frequency, the most effective herbs included: Baizhu, Huangqi, Fuling, Bai Hua She She Cao, Ezhu, Dangshen, and Gancao. The compounds of seven herbs were as follows: *Atractylodes macrocephala* Koidz. (Asteraceae, Atractylodis Macrocephalae rhizoma); *Astragalus mongholicus* Bunge (Fabaceae, Astragali radix); *Smilax glabra* Roxb. (Smilacaceae, Rhizoma smilacis glabrae); *Scleromitrion diffusum* (Willd.) R. J. Wang (Rubiaceae, *Oldenlandiae diffusae herba*); *Curcuma aromatica* Salisb. (Zingiberaceae, Curcumae Longae Radix); *Codonopsis pilosula* (Franch.) Nannf. (Campanulaceae, Codonopsis pilosulae radix); and *Glycyrrhiza glabra* L. (Fabaceae, Extractum glycyrrhizae), as shown in Table 3. A new effective formula was made up of these seven herbs for network pharmacology analysis.

**TABLE 3:** High-frequency Chinese herbs.

Pharmaceutical name	Chinese name	Counts	Frequency 1 (counts/total herb counts)	Frequency 2 (counts/study numbers	(%) Class of natural compound
*Atractylodes macrocephala* Koidz	Baizhu	19	7.22	79.17	Asteraceae; Atractylodis macrocephalae rhizoma
*Astragalus mongholicus* Bunge	Huangqi	17	6.46	70.83	Fabaceae; Astragali radix
*Smilax glabra* Roxb.	Fuling	15	5.70	62.50	Smilacaceae; Rhizoma smilacis glabrae
*Scleromitrion diffusum* (Willd.) R.J.Wang	Bai Hua She She Cao	14	5.32	58.33	Rubiaceae; *Oldenlandiae diffusae herba*
*Curcuma aromatica* Salisb.	Ezhu	14	5.32	58.33	Zingiberaceae; Curcumae Longae Radix
*Codonopsis pilosula* (Franch.) Nannf	Dangshen	12	4.56	50.00	Campanulaceae; Codonopsis pilosulae radix
*Glycyrrhiza glabra* L	Gancao	12	4.56	50.00	Fabaceae; Extractum glycyrrhizae

#### Establishment of herb–component–target network

**FIGURE 12:**
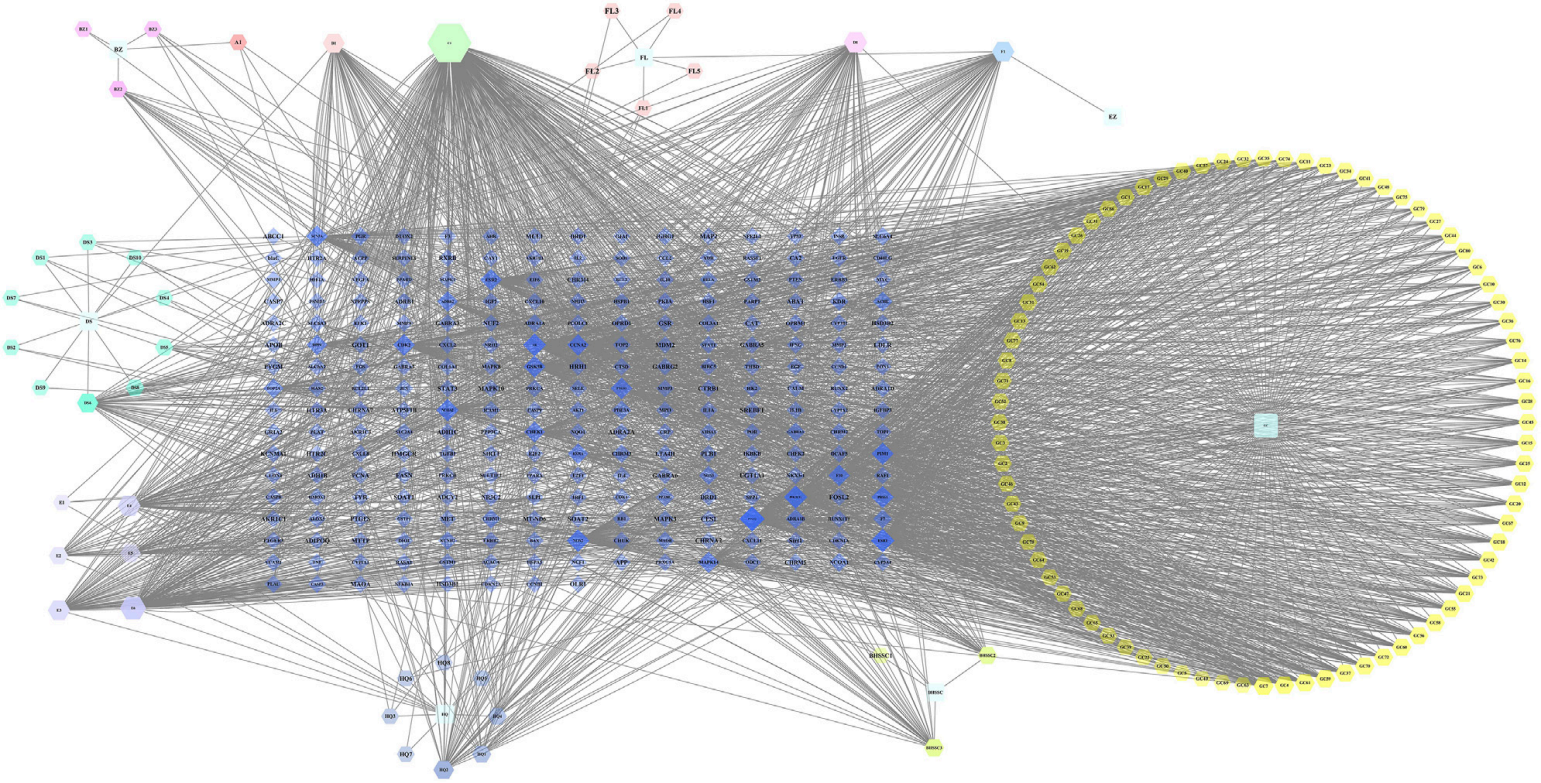
Network of Herb-Component-Target.

Through the search of the prescribed database, the seven effective herbs were found to be comprised of 120 compounds, and 246 herb target genes. Cytoscape3.7.2 software was adopted to establish the network of the compound and target genes of the effective herbs (Figure 12).

#### Screening of intersectional genes of effective herbs, AOC, and the PPI network

**FIGURE 13:**
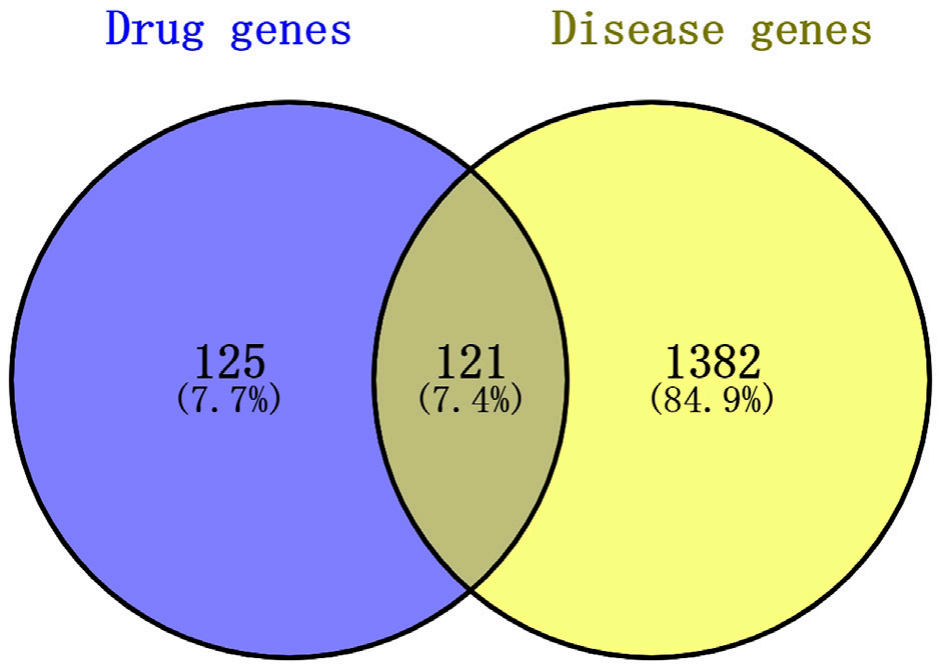
The number of intersection targets of effective herbs and AOC.

**FIGURE 14:**
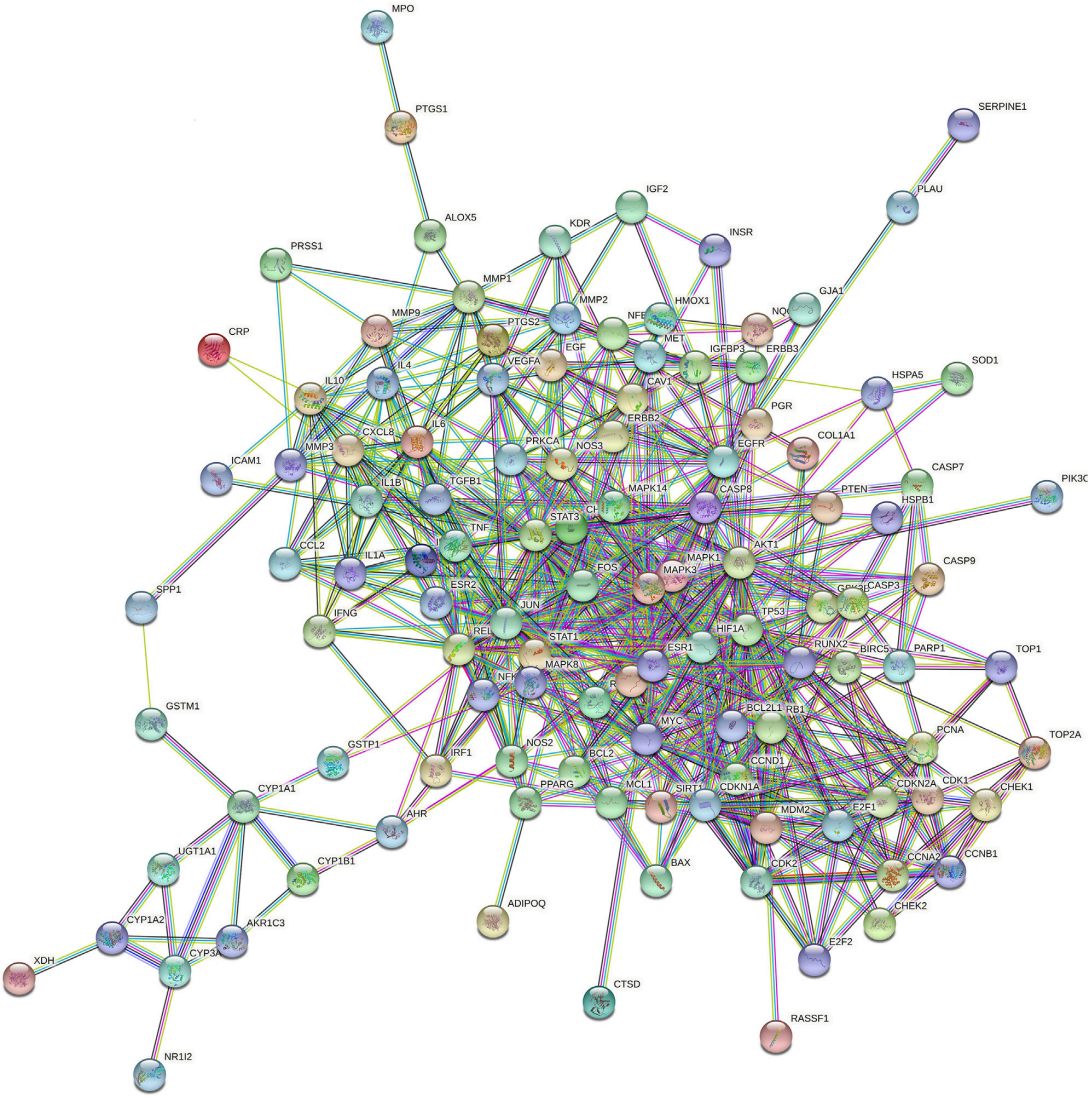
The PPI network of the intersection genes of herb targets and disease targets.

**FIGURE 15:**
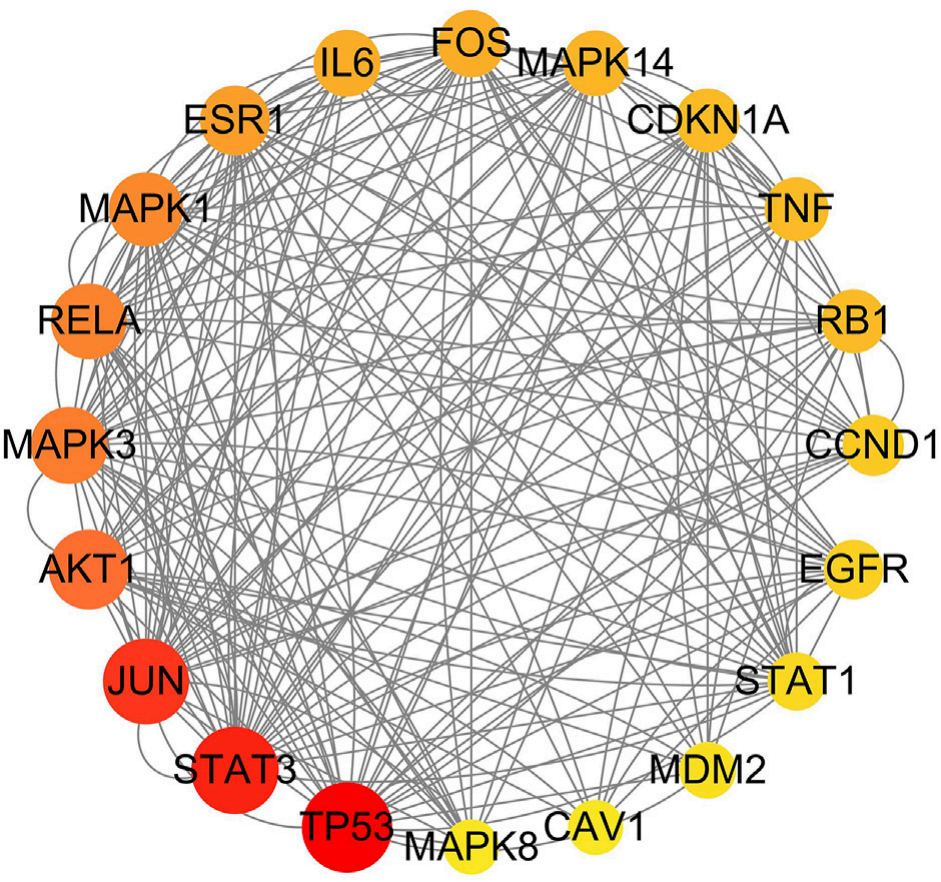
The 20 core genes from the intersection genes.

According to the four databases, AOC had 1503 disease targets: 121 target genes of herbs and disease intersected through the Venn diagram (Figure 13). The intersecting genes of the herb targets and the AOC targets were mapped into the STRING database, and the PPI network obtained. The network contained 121 nodes and 618 edges, with the average node degree being 10.2 (*p* < 1.0e-16) (Figure 14). The PPI network was introduced into Cytoscape, in which the CytoNCA plug-in was used and the first 20 genes were extracted as core genes (Figure 15).

#### GO and KEGG enrichment analysis

From the analysis of Supplementary Figure S1, the GO terms of the BP were mainly related to response to inorganic substances, response to reactive oxygen species, response to xenobiotic stimulus, response to oxidative stress, positive regulation of cellular component movement, positive regulation of cell migration, gland development, cellular response to chemical stress, positive regulation of cell motility, and response to hormones. The CC was mainly related to the transcription regulator complex, membrane raft, membrane microdomain, vesicle lumen, cyclin-dependent protein kinase holoenzyme complex, protein kinase complex, RNA polymerase II transcription regulator complex, secretory granule lumen, cytoplasmic vesicle lumen, and the serine/threonine protein kinase complex. In addition, the GO terms of the MF were mainly related to DNA-binding transcription factor binding, transcription factor binding, kinase binding, RNA polymerase II-specific DNA-binding transcription factor binding, protein kinase binding, protein kinase activity, phosphotransferase activity, alcohol group as acceptor, protein domain specific binding, kinase activity, and ubiquitin-like protein ligase binding.

The top 20 KEGG pathways are shown in Supplementary Figure S2. This verifies pathways in cancer (hsa05200), prostate cancer (hsa05215), bladder cancer (hsa05219), pancreatic cancer (hsa05212), the PI3K-Akt signaling pathway (hsa04151), proteoglycans in cancer (hsa05205), and hepatocellular carcinoma (hsa05225). The target–pathway network was constructed with the outer circle as the core gene and the inner circle as the related pathway according to the results of the KEGG enrichment analysis (Supplementary Figure S3).

## Discussion

With changes in life pressure and dietary structure, the incidence of OC is increasing. Because of non-obvious early symptoms, OC has the characteristic of a low early diagnosis rate, and more than 70% of patients are in advanced stages when diagnosed (Zhu et al., 2016). AOC develops rapidly, and tumor cells can spread to the uterus, omentum, and other parts in a short time, increasing the difficulty of treatment. Numerous studies have shown that Chinese herbs can effectively relieve the clinical symptoms of AOC patients and play an important role in the treatment of AOC. In a narrow sense, Chinese herbs refers to plant medicine. The medicinal part is divided into root and rhizome, stem and wood, bark, leaf, flower, fruit and seed, and whole herb, etc., and excludes animal medicine such as leech, and mineral medicine such as keel. However, in a broad sense, Chinese herbs can be understood as all non-proprietary Chinese medicines (Wang, 2020). To explore the efficacy and safety of Chinese herbs in the treatment of AOC and its effective targets, we used a method of integrating meta-analysis and network pharmacology analysis.

These meta-analysis results reveal that, compared with chemotherapy alone, the treatment of TCM combined with chemotherapy improved the ORR of AOC patients. In solid tumor treatment, the ORR indicates the sum of patients with complete and partial remission after treatment in relation to the total number of evaluable cases, which is equal to the sum of cases in complete remission and partial remission divided by the total number of evaluable cases. TCM can also improve the 3-year survival rate and PFS, which illustrates its efficacy in increasing the sensitivity of chemotherapy, and avoiding the chemo-resistance and experience of relapses in the treatment of AOC. However, TCM had no significant effect on improving the 5-year survival rate in the analysis, although in the original study of Chen (2012), the 5-year survival rate of TCM combined with chemotherapy in the treatment of AOC was 55.6%, while the survival rate of chemotherapy alone was 40.0%, which suggests that integrated TCM and chemotherapy can improve the 5-year survival rate. However, only one study reported this indicator, which may lead to bias, and hence more cases are needed to illustrate this outcome. As for adverse events, our meta-analysis showed that TCM can significantly reduce the incidence of gastrointestinal reactions, including nausea, diarrhea, and constipation caused by chemotherapy, since high frequency herbs, such as Baizhu, Huangqi, and Dangshen, function to tonify spleen and stomach, etc. Moreover, one study pointed out that TCM can restore intestinal mucosal epithelial cells, tight junctions, and protect the permeability of the intestinal mucosa barrier in rats (Shi et al., 2017). It therefore has efficacy in reducing gastrointestinal reactions. In addition, TCM has a positive impact in reducing myelosuppression, that is, leukopenia, anemia, and thrombocytopenia. In addition, TCM can reduce the occurrence of liver and kidney damage. TCM has anti-oxidative and anti-inflammatory effects on liver diseases, which may be its mechanism in reducing the occurrence of liver damage (Lam et al., 2016). These potential mechanisms of TCM on renal injury include anti-inflammation, antioxidative effect, anti-cell death, and regulation of the energy metabolism by restoring Na + -K + -ATPase activity etc. (Liu et al., 2021). Due to the limited number of included studies, only some other adverse effects were reduced as a result of treatment with TCM, and more related studies are needed to further verify these. All in all, TCM combined with chemotherapy for AOC patients is safer than chemotherapy alone, and can reduce the incidence of adverse reactions.

After the meta-analysis was completed, we extracted high-frequency herbs from all the TCM prescriptions in the included studies of effective herbs. Their composition was Baizhu, Huangqi, Fuling, Bai Hua She She Cao, Ezhu, Dangshen, and Gancao. In TCM theory, OC belongs to “Zhengjia,” the root cause of which is the declining function of the spleen and stomach qi, leading to deficiency of qi and blood. Blood stasis in the ovarian area is another important pathogenesis. The effective herbs of Baizhu, Fuling, Dangshen, and Gancao are a Sijunzi decoction, which is good at replenishing qi and invigorating the spleen function. Huangqi is also a qi-invigorating herb that can enhance the efficacy of a Sijunzi decoction, and the above 5 herbs can tonify qi and help the body expel pathogens. The functions of Ezhu are breaking blood and activating qi, removing stagnation, and relieving pain, which is suitable for the pathogenesis of blood stasis. Bai Hua She She Cao is good at clearing away heat and toxic materials as well as reducing swelling and removing stasis, and has been confirmed as a key antitumor herb in many studies. These seven effective herbs are mutually compatible, achieving the effect of strengthening the healthy qi and anti-tumor effect.

We also performed network pharmacology to explore the specific effects of effective herbs on AOC. We screened 120 components and 246 targets of effective herbs and constructed the PPI network by integrating 121 intersecting targets of these seven herbs associated with AOC. Among the herb–component–target network, the 10 most important components were selected according to the degree value, including MOL000098, MOL000422, MOL003896, MOL000392, MOL000354, MOL000296, MOL000449, MOL000006, MOL000378, and MOL000417, including: quercetin, kaempferol, 7-methoxy-2-methyl isoflavone, formononetin, isorhamnetin, hederagenin, stigmasterol, luteolin, 7-O-methylisomucronulatol, and calycosin. Considerable evidence supports the anti-tumor function of the aforementioned components of kaempferol, quercetin, formononetin, and isorhamnetin (Rauf et al., 2018; Imran et al., 2019; Zhang et al., 2019; Cai et al., 2020).

In addition, the 20 core genes in the PPI networks were *TP53*, *STAT3*, *JUN*, *AKT1*, *MAPK3*, *RELA*, *MAPK1*, *ESR1*, *IL6*, *FOS*, *MAPK14*, *TNF*, *CDKN1A*, *RB1*, *CCND1*, *EGFR*, *STAT1*, *MDM2*, *MAPK8*, and *CAV1*. Clinical studies have shown that the above core genes are related to the occurrence and development of a variety of tumors. For example, *TP53* gets activated in response to a variety of stress signals, such as DNA damage, and hyperproliferative signals, and is involved in the orchestration of basic events that must be overcome for cancer initiation and progression (Bieging et al., 2014). *STAT3* cooperates with other targets in promoting glycolysis or lipid catabolism, which have potential roles in different aspects of the metabolism switches in cancer cells that support tumor progression (Martincuks et al., 2020). *AKT1* is a direct target of miR-153 in ovarian cancer cells (Li et al., 2017). Next, according to the results of GO enrichment analysis, we found that effective herbs exert a therapeutic effect on AOC mainly through response to reactive oxygen species, response to oxidative stress, and positive regulation of cell migration, which are all closely related to cancer (Prasad et al., 2017; Klaunig, 2018). KEGG enrichment analysis showed that there are many pathways directly related to different types of tumors, such as the pathway in cancer, prostate cancer, bladder cancer, and pancreatic cancer. Other pathways, such as the PI3K/AKT signaling pathway, are also closely associated with the occurrence of cancer (Ma et al., 2020). Hence, the aforementioned pathways play an important role in treating AOC, through which the components of effective herbs may achieve the desired effect.

This review has several limitations. First, the quality evaluation of many articles in terms of allocation concealment and blinding was unclear, and the lack of large, multicenter RCTs may lead to the potential risk of bias and affect the reliability of the results. Second, the differences in application of chemotherapy and duration of treatment among the included trials may lead to a certain degree of heterogeneity. Third, screening of herb components based on DL and OB values may miss some effective components.

Despite these limitations, this study is the first to integrate meta-analysis and network pharmacology to explore the efficacy and potential pharmacological mechanisms of TCM on AOC. We hope it will provide evidence for clinicians to treat AOC patients with a better strategy, as well as provide scientific clues for researchers in this field, which can be further validated experimentally.

## Conclusion

In conclusion, this article reveals that combined with chemotherapy, TCM is more effective and safer than chemotherapy alone in treating AOC. In addition, TCM treats AOC patients through a multi-target, multi-component, and multi-pathway mechanism. To make these results more reliable, more rigorously designed RCTs are required in the future and further pharmacological experiments *in vivo* and *in vitro* are needed to validate the therapeutic mechanism of these findings.

## Data Availability

The original contributions presented in the study are included in the article/Supplementary Material; further inquiries can be directed to the corresponding authors.

## Supplementary material

The supplementary material for this article can be found online at: https://www.frontiersin.org/articles/10.3389/fphar.2022.1040641/full#supplementary-material

Supplementary Figure S1 GO enrichment analysis.

Supplementary Figure S2 KEGG enrichment analysis.

Supplementary Figure S3 Network of target–pathway.

## References

[B1] Bieging K. T. Mello S. S. Attardi L. D. (2014). Unravelling mechanisms of p53-mediated tumour suppression. Nat. Rev. Cancer 14 (5), 359–370. 10.1038/nrc3711 24739573 PMC4049238

[B2] Cai F. Zhang Y. Li J. Huang S. Gao R. (2020). Isorhamnetin inhibited the proliferation and metastasis of androgen-independent prostate cancer cells by targeting the mitochondrion-dependent intrinsic apoptotic and PI3K/Akt/mTOR pathway. Biosci. Rep. 40 (3), BSR20192826. 10.1042/bsr20192826 32039440 PMC7080645

[B3] Carioli G. Malvezzi M. Bertuccio P. Boffetta P. Levi F. La Vecchia C. (2021). European cancer mortality predictions for the year 2021 with focus on pancreatic and female lung cancer. Ann. Oncol. 32 (4), 478–487. 10.1016/j.annonc.2021.01.006 33626377

[B4] Chen J. (2012). Clinical efficacy evaluation and survival analysis of integrated traditional Chinese and Western medicine in the treatment of advanced ovarian cancer. Zhejiang J. Traditional Chin. Med. 47 (10), 751–752. 10.3969/j.issn.0411-8421.2012.10.040

[B5] Chen S. Hua Y. (2017). Therapeutic effect of warming yang and nourishing qi herbs assisted by hyperthermic peritoneal perfusion Ovarian cancer secondary to peritoneal effusion. Chin. Med. Emerg. 26 (08), 1487–1489. 10.3969/j.issn.1004-745X.2017.08.055

[B6] Chen Z. Ren H. Peng T. (2014). Clinical observation of Fuzheng Xiaoliu decoction combined with paclitaxel in the treatment of advanced ovarian cancer. Oncol. Pharm. 4 (03), 226–228. 10.3969/j.issn.2095-1264.2014.045

[B7] Colombo N. Sessa C. du Bois A. Ledermann J. McCluggage W. G. McNeish I. (2019). ESMO-ESGO consensus conference recommendations on ovarian cancer: Pathology and molecular biology, early and advanced stages, borderline tumours and recurrent disease. Ann. Oncol. 30 (5), 672–705. 10.1093/annonc/mdz062 31046081

[B8] Craig A. D. Garcia E. Peters P. N. Chen L. M. Chapman J. S. (2021). Primary treatment of advanced ovarian cancer: How does the 'real world' practice? Future Oncol. 17 (34), 4687–4696. 10.2217/fon-2021-0086 34435878

[B9] Cumpston M. Li T. Page M. J. Chandler J. Welch V. A. Higgins J. P. (2019). Updated guidance for trusted systematic reviews: A new edition of the Cochrane Handbook for systematic reviews of interventions. Cochrane Database Syst. Rev. 10, Ed000142. 10.1002/14651858.Ed000142 31643080 PMC10284251

[B10] Dai S. Liu K. (2021). Clinical effect and safety of Guizhi Fuling Pill in adjuvant treatment of advanced ovarian cancer. J. Clin. Ration. Drug Use 14 (23), 145–147. 10.15887/j.cnki.13-1389/r.2021.23.060

[B11] Fang Y. Xing W. Wang W. (2019). Effects of Wenyang Yiqi Jianpi Decoction combined with chemotherapy on immune function and serum HE4 and CA125 levels in patients with advanced ovarian cancer. Chin. J. Traditional Chin. Med. 34 (06), 2819–2822.

[B12] Han F. Shen Y. (2015). On the intermediary mechanism in micro-factors between Chinese medicine constitutions and ovarian cancer. Shanghai J. Traditional Chin. Med. 49 (12), 8–10. 10.16305/j.1007-1334.2015.12.003

[B13] Hou X. Wu X. (2018). To investigate the effect of self-made traditional Chinese medicine prescription combined with TP regimen on the immune function and survival of advanced ovarian cancer patients. Appl. Mod. Med. China 12 (08), 96–98. 10.14164/j.cnki.cn11-5581/r.2018.08.052

[B14] Hu H. (2019). Clinical efficacy of Xiaodu Yiai Decoction combined with TC regimen in the treatment of advanced ovarian cancer and its relationship with the level of T cell subsets. J. Hubei Univ. Traditional Chin. Med. 21 (06), 26–29. 10.3969/j.issn.1008987x.2019.06.06

[B15] Huang W. Gao Y. Jiao Z. (2022). Effect of Wenyang Yiqi Jianpi Decoction combined with chemotherapy on the efficacy and immune function, serum HE4 and CA125 levels in patients with advanced ovarian cancer. New Chin. Med. 54 (09), 139–142. 10.13457/j.cnki.jncm.2022.09.032

[B16] Imran M. Salehi B. Sharifi-Rad J. Aslam Gondal T. Saeed F. Imran A. (2019). Kaempferol: A key emphasis to its anticancer potential. Molecules 24 (12), E2277. 10.3390/molecules24122277 PMC6631472 31248102

[B17] Jia F. (2017). Analysis of the effect of self-made traditional Chinese medicine prescription combined with conventional Western medicine chemotherapy in the treatment of advanced ovarian cancer. Strait Pharm. 29 (02), 169–171. 10.3969/j.issn.1006-3765.2017.02.093

[B18] Jin W. Kong C. (2015). Fuzheng Xiaoliu decoction combined with paclitaxel in the treatment of 43 cases of advanced ovarian cancer. Henan Tradit. Chin. Med. 35 (12), 3122–3123. 10.16367/j.issn.1003-5028.2015.12.1343

[B19] Klaunig J. E. (2018). Oxidative stress and cancer. Curr. Pharm. Des. 24 (40), 4771–4778. 10.2174/1381612825666190215121712 30767733

[B20] Lam P. Cheung F. Tan H. Y. Wang N. Yuen M. F. Feng Y. (2016). Hepatoprotective effects of Chinese medicinal herbs: A focus on anti-inflammatory and anti-oxidative activities. Int. J. Mol. Sci. 17 (4), 465. 10.3390/ijms17040465 27043533 PMC4848921

[B21] Lheureux S. Braunstein M. Oza A. M. (2019). Epithelial ovarian cancer: Evolution of management in the era of precision medicine. Ca. Cancer J. Clin. 69 (4), 280–304. 10.3322/caac.21559 31099893

[B22] Lheureux S. Gourley C. Vergote I. Oza A. M. (2019). Epithelial ovarian cancer. Lancet 393 (10177), 3931240–3931253. 10.1016/s0140-6736(18)32552-2 30910306

[B23] Li W. Wang M. Meng B. Yu J. Chen Q. Li H. (2017). MicroRNA-153 regulated AKT1 expression and suppressed cell proliferation of epithelial ovarian cancer cells. Int. J. Clin. Exp. Pathol. 10 (7), 7417–7426. 31966584 PMC6965221

[B24] Li Y. Li J. Fan B. Wang Y. Jiang J. Zhang Z. (2020). Efficacy and safety of Yiqi Huoxue Jiedu decoction for the treatment of advanced epithelial ovarian cancer patients: A double-blind randomized controlled clinical trial. J. Tradit. Chin. Med. 40 (1), 103–111. 10.19852/j.cnki.jtcm.2020.01.011 32227771

[B25] Li C. Zhu P. Yu J. Liu J. Liu Y. (2021). Efficacy of Yiqi Huoxue Jiedu Decoction combined with chemotherapy in the treatment of advanced ovarian cancer and its effect on immune pathway-related target genes. Shaanxi Tradit. Chin. Med. 42 (08), 1072–1075+1079. 10.3969/j.issn.1000-7369.2021.08.021

[B26] Liang R. (2013). Effects of adjuvant therapy with Zengmai Yiliu Decoction on the adverse reactions of postoperative chemotherapy and the quality of life of patients with advanced ovarian cancer. Chin. J. Traditional Chin. Med. 31 (11), 2588–2590. 10.13193/j.issn.1673-7717.2013.11.082

[B27] Liu D. Tang S. Gan L. Cui W. (2021). Renal-protective effects and potential mechanisms of traditional Chinese medicine after ischemia-reperfusion injury. Evid. Based. Complement. Altern. Med. 2021, 5579327. 10.1155/2021/5579327 PMC7910071 33680054

[B28] Ma Z. Lou S. Jiang Z. (2020). PHLDA2 regulates EMT and autophagy in colorectal cancer via the PI3K/AKT signaling pathway. Aging (Albany NY) 12 (9), 7985–8000. 10.18632/aging.103117 32385195 PMC7244065

[B29] Martincuks A. Li P. C. Zhao Q. Zhang C. Li Y. J. Yu H. (2020). CD44 in ovarian cancer progression and therapy resistance-A critical role for STAT3. Front. Oncol. 10, 589601. 10.3389/fonc.2020.589601 33335857 PMC7736609

[B30] Menon U. Karpinskyj C. Gentry-Maharaj A. (2018). Ovarian cancer prevention and screening. Obstet. Gynecol. 131 (5), 909–927. 10.1097/aog.0000000000002580 29630008

[B31] Pan J. (2018). Yiqi Jianpi Yangxue Decoction combined with docetaxel and cisplatin in the treatment of advanced ovarian cancer. Chin. Med. J. 33 (07), 1186–1189. 10.16368/j.issn.1674-8999.2018.07.281

[B32] Pei X. (2010). Clinical study on the treatment of ovarian cancer with modified Lichong decoction combined with chemotherapy [硕士]. Cnki: Xingjiang Medical College.

[B33] Prasad S. Gupta S. C. Tyagi A. K. (2017). Reactive oxygen species (ROS) and cancer: Role of antioxidative nutraceuticals. Cancer Lett. 387, 95–105. 10.1016/j.canlet.2016.03.042 27037062

[B34] Rauf A. Imran M. Khan I. A. Ur-Rehman M. Gilani S. A. Mehmood Z. (2018). Anticancer potential of quercetin: A comprehensive review. Phytother. Res. 32 (11), 2109–2130. 10.1002/ptr.6155 30039547

[B35] Ren J. Feng J. (2019). Effects of Fuzheng Quji Decoction on serum tumor markers and clinical efficacy in patients with advanced ovarian cancer. Chin. Cancer Clin. Rehabilitation 26 (07), 844–847. 10.13455/j.cnki.cjcor.2019.07.21

[B36] Shi L. Wang J. Yang Q. Shi L. Liu L. Feng X. (2017). Effect of Yang-activating and stasis-eliminating decoction from Traditional Chinese Medicine on intestinal mucosal permeability in rats with ulcerative colitis induced by dextran sulfate sodium. J. Traditional Chin. Med. 37 (4), 452–460. 10.1016/s0254-6272(17)30151-6 32188203

[B37] Torre L. A. Trabert B. DeSantis C. E. Miller K. D. Samimi G. Runowicz C. D. (2018). Ovarian cancer statistics. Ca. Cancer J. Clin. 68 (4), 284–296. 10.3322/caac.21456 29809280 PMC6621554

[B38] Wang D. Yang G. Wang Y. Ayia M. Pang R. (2016). Correlation between TCM constitution types and TCM syndrome types in patients with advanced ovarian cancer. J. Mod. Integr. Med. 25 (17), 1866–1867. 10.3969/j.issn.1008-8849.2016.17.014

[B39] Wang N. Xiao F. Shao H. Shi S. Zhou Y. (2022). Clinical efficacy of Yiqi Yangyin decoction combined with docetaxel on advanced ovarian cancer and the effect on the levels of serum markers VEGF, HE4, and CA125. J. Healthc. Eng. 2022, 8401202. 10.1155/2022/8401202 35368946 PMC8967517

[B40] Wang S. (2020). Research and application of Knowledge extraction meth of Chinese herb literature. [master]. Cnki: Jilin University.

[B41] Xu X. Zhou C. Wang J. Jia J. Chen X. (2015). Effects of postoperative chemotherapy regimens on patients with advanced ovarian cancer on serum alpha-fetoprotein, HE4 and CA125 levels. J. Clin. Ration. Drug Use 8 (07), 102–103. 10.15887/j.cnki.13-1389/r.2015.07.062

[B42] Yang L. (2017). Effect of Huoxue Jiedu Decoction on the curative effect and serum inflammatory factors in patients with advanced ovarian cancer. Med. Inf. 30 (3), 176–177. chi. 10.3969/j.issn.1006-1959.2017.03.119

[B43] Yang L. (2020). Effects of applying TP regimen combined with hyperthermic perfusion chemotherapy on immune function and specific indexes of serum in elderly patients with advanced ovarian cancer. Nurs. Pract. Res. 17 (04), 116–117. 10.3969/j.issn.1672-9676.2020.04.044

[B44] Yang W. Mi X. (2021). Efficacy of compound Daqiqi decoction combined with TC regimen in the treatment of advanced ovarian cancer and its effect on serum B7-H4 and HE4 levels. Sichuan Tradit. Chin. Med. 39 (08), 153–156.

[B45] Zhang J. (2016). Efficacy of self-made traditional Chinese medicine decoction on patients with advanced ovarian cancer and its effect on immune function and tumor markers. Int. Med. health Rep. 22 (17), 2691–2694. 10.3760/cma.j.issn.1007-1245.2016.17.037

[B46] Zhang C. Zeng S. Wang L. (2018). Influence of integrated traditional Chinese and Western medicine treatment on immunology and other indicators and survival rate of patients with advanced ovarian cancer. Bright Chin. Med. 33 (15), 2248–2250. 10.3969/j.issn.1003-8914.2018.15.046

[B47] Zhang Y. Chen C. Zhang J. (2019). Effects and significance of formononetin on expression levels of HIF-1α and VEGF in mouse cervical cancer tissue. Oncol. Lett. 18 (3), 2248–2253. 10.3892/ol.2019.10567 31452725 PMC6676657

[B48] Zhao A. Zhao S. Wang F. (2016). Observation on the curative effect of Taohong Siwu decoction combined with chemotherapy in the treatment of ovarian cancer. Med. Inf. 29 (17), 333–334. 10.3969/j.issn.1006-1959.2016.17.316

[B49] Zhou G. (2017). Clinical effect of Jianpi Jiedu Sanjie Recipe combined with TP regimen in the treatment of advanced ovarian cancer. Traditional Chin. Med. Clin. Res. 9 (05), 118–119. 10.3969/j.issn.1674-7860.2017.05.061

[B50] Zhou Q. Zhou F. Zhang X. (2020). Clinical study of modified Lichong decoction combined with TC regimen in the treatment of advanced ovarian cancer. New Chin. Med. 52 (09), 39–43. 10.13457/j.cnki.jncm.2020.09.011

[B51] Zhu S. Tan J. Zhang C. Wu Q. Xie X. Yin H. (2016). Effects of Lipusu combined with nedaplatin chemotherapy on the changes of serum HE4, CA125, CA19-9, AFP, CEA and T cell subsets in patients with advanced ovarian cancer. J. Hainan Med. Coll. 22 (15), 1737–1740. 10.13210/j.cnki.jhmu.20160421.007

