# Supplementary figures and images for: The pharmacological mechanism of Chinese herbs effective in treating advanced ovarian cancer: Integrated meta-analysis and network pharmacology analysis
Source: Front Pharmacol. 2022 Nov 9;13:1040641. doi: 10.3389/fphar.2022.1040641 (PMC9682081; doi:10.3389/fphar.2022.1040641)

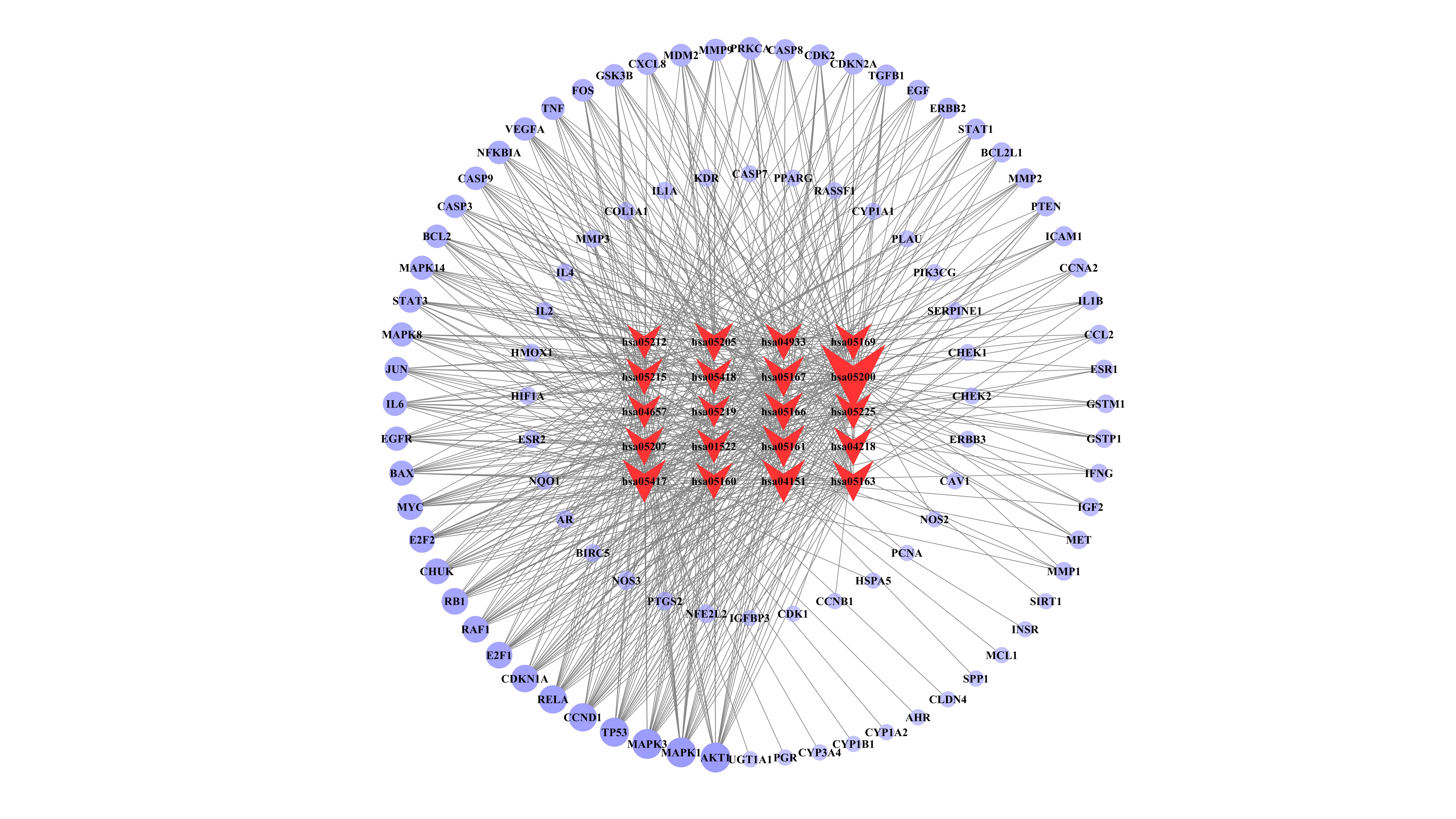

Supplement: Supplementary file 1 [file Image3.JPEG]

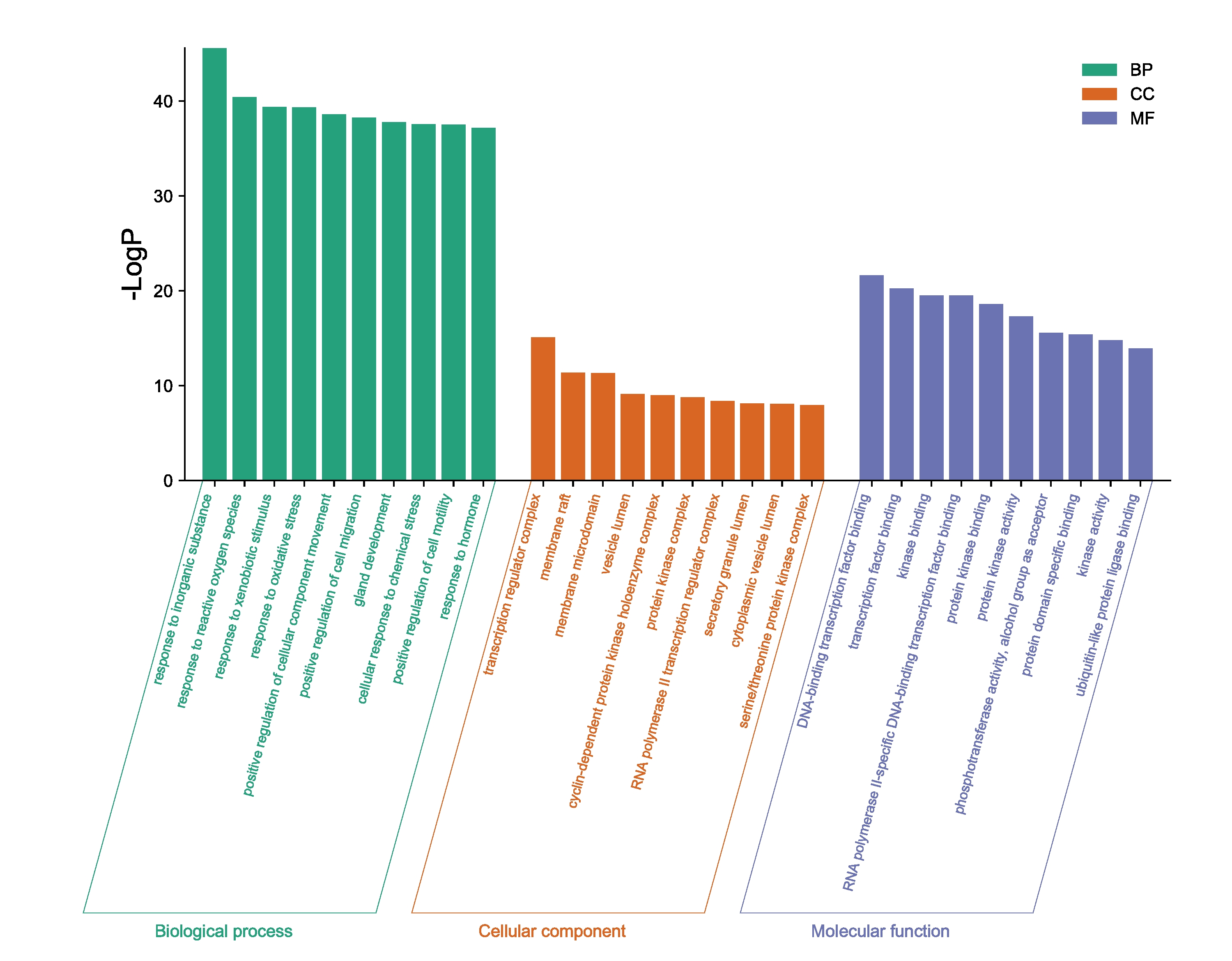

Supplement: Supplementary file 2 [file Image1.JPEG]

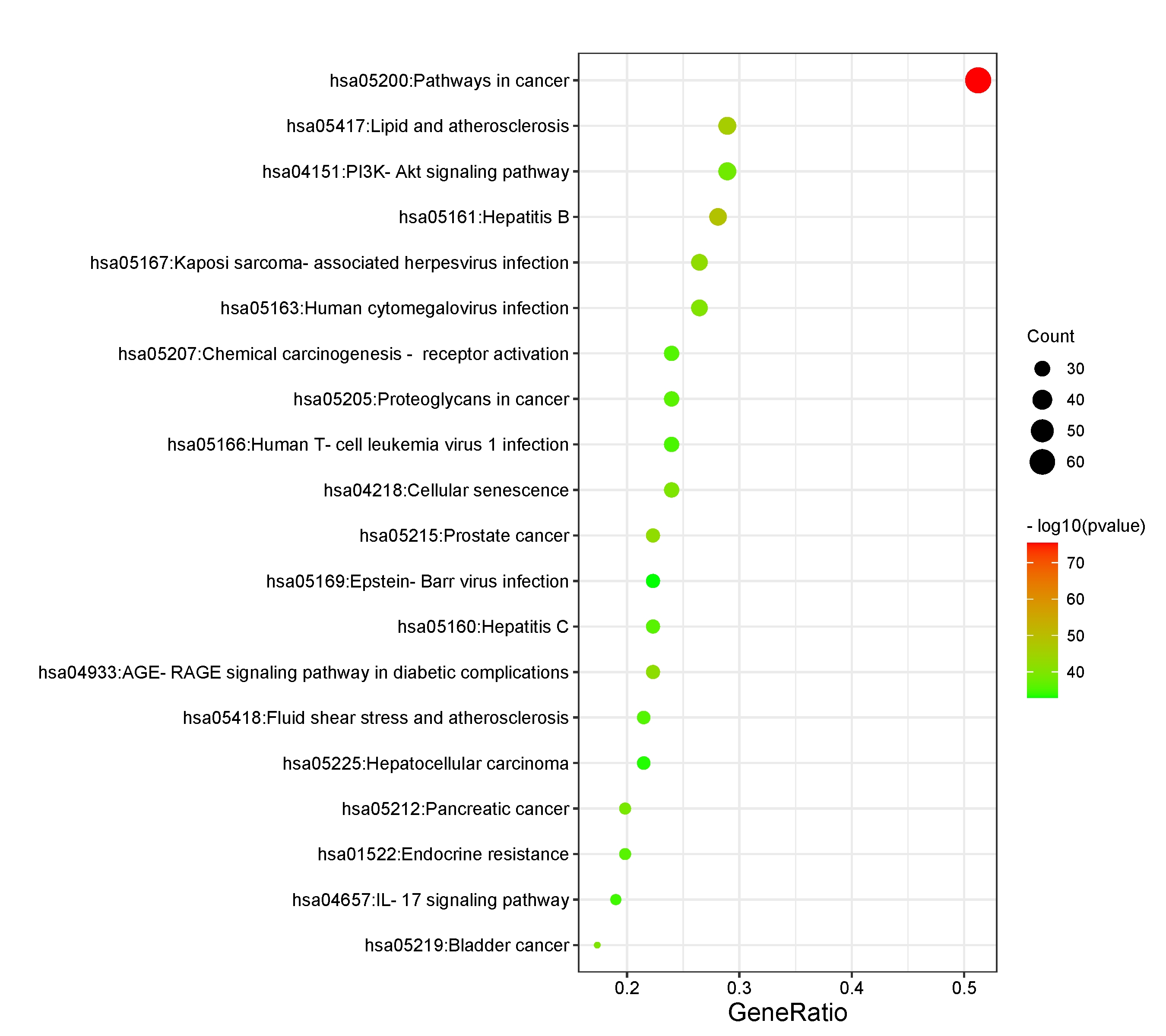

Supplement: Supplementary file 3 [file Image2.JPEG]
